# Intermuscular Coherence in Normal Adults: Variability and Changes with Age

**DOI:** 10.1371/journal.pone.0149029

**Published:** 2016-02-22

**Authors:** Stephan R. Jaiser, Mark R. Baker, Stuart N. Baker

**Affiliations:** 1 Institute of Neuroscience, Medical School, Newcastle University, Newcastle upon Tyne, United Kingdom; 2 Department of Neurology, Royal Victoria Infirmary, Newcastle upon Tyne, United Kingdom; 3 Department of Clinical Neurophysiology, Royal Victoria Infirmary, Newcastle upon Tyne, United Kingdom; University Medical Center Groningen UMCG, NETHERLANDS

## Abstract

We investigated beta-band intermuscular coherence (IMC) in 92 healthy adults stratified by decade of age, and analysed variability between and within subjects. In the dominant upper limb, IMC was estimated between extensor digitorum communis and first dorsal interosseous as well as between flexor digitorum superficialis and first dorsal interosseous. In the ipsilateral lower limb, IMC was measured between medial gastrocnemius and extensor digitorum brevis as well as between tibialis anterior and extensor digitorum brevis. Age-related changes in IMC were analysed with age as a continuous variable or binned by decade. Intrasession variance of IMC was examined by dividing sessions into pairs of epochs and comparing coherence estimates between these pairs. Eight volunteers returned for a further session after one year, allowing us to compare intrasession and intersession variance. We found no age-related changes in IMC amplitude across almost six decades of age, allowing us to collate data from all ages into an aggregate normative dataset. Interindividual variability ranged over two orders of magnitude. Intrasession variance was significantly greater than expected from statistical variability alone, and intersession variance was even larger. Potential contributors include fluctuations in task performance, differences in electrode montage and short-term random variation in central coupling. These factors require further exploration and, where possible, minimisation. This study provides evidence that coherence is remarkably robust to senescent changes in the nervous system and provides a large normative dataset for future applications of IMC as a biomarker in disease states.

## Introduction

Beta-band (15-30Hz) oscillations are demonstrable in the motor systems of monkeys [1] and humans [2–4]. During sustained contractions, these oscillations are coherent between sensorimotor cortex and contralateral muscles (corticomuscular coherence, CMC) [4–7], between co-contracting muscles within one limb (intermuscular coherence, IMC) [6] and between single motor units in one muscle (intramuscular coherence, IntraMC) [8]. Whilst measured between different pairs of signals, CMC, IMC and IntraMC reflect the same central coupling mechanism. Although often deemed a purely efferent phenomenon mediated by the corticospinal tract [9], early suggestions that beta-band coherence also depends on afferent pathways [5] have now been substantiated and imply an underlying efferent-afferent feedback loop [10,11].

Beta-band CMC [12,13] and IMC [14] are absent in infancy and develop during the early teenage years alongside the rising capacity for fractionated movement [15]. Although the corticospinal tract establishes functional projections before birth [16] and becomes fully myelinated by two years of age [17], development only completes during adolescence [14] when intracortical inhibition increases [18]. Computational models suggest that inhibitory mechanisms are critical to oscillatory activity [19] and thus to coherence.

Senescent changes in coherence are controversial. One study reported that beta-band CMC shows a single peak at 23Hz in young adults which is replaced by multiple peaks at lower or higher frequencies in the elderly [13]. Other reports described age-related decreases in the peak frequency of CMC [20] or IntraMC [21]. Peak coherence amplitude was reported to remain unchanged [13,21] or to increase with age [20]. These discrepancies are not easily reconciled. Motor function is known to deteriorate in old age [22–24]. Several potential substrates have been proposed, including decreases in neuronal size [25], dendritic arborisation [26] and synaptic density [27] in the cortical grey matter, reduced intracortical inhibition [28], diminished white matter volume [29] with leukoaraiosis on MRI [30], a decline in peripheral motor and sensory conduction velocities [31,32] and remodelling of motor units [33]. Whether coherence is sensitive to these changes is unclear, although it could plausibly be affected by decreased intracortical inhibition and prolonged latencies in the efferent-afferent loop.

Only two studies have investigated the variability of coherence within subjects. One compared pairs of CMC measurements from either the same session (‘intrasession’) or two sessions separated by one year (‘intersession’) in ten subjects [34]. Between intrasession measurements, frequency and amplitude of the 15-30Hz CMC peak were each strongly correlated. Equivalent intersession correlations were weaker, with coherence amplitude varying by 17–220% in individual subjects. Unfortunately, correlation analysis constitutes a suboptimal approach as it disregards known distributional properties of intraindividual differences in coherence. In one subject, CMC was estimated eight times over 20 months, demonstrating relative constancy of peak CMC frequency but marked variation in amplitude. A second study assessed CMC three times in one subject over two years [11]. Peak CMC amplitude varied substantially, alongside changes in the directionality of CMC within the efferent-afferent loop.

It has recently been shown that beta-band IMC constitutes a potential biomarker of corticospinal tract function [35]. Here, we sought to define age-related changes in IMC in a large, age-stratified adult sample, and to gather a normative dataset for future comparison against data from disease states. In a subset, we analysed the variability of IMC within and between sessions using dedicated statistical methods.

We found that beta-band IMC did not change significantly across adulthood, and was highly variable between subjects. For a given subject and session, variability was larger than statistically expected, with even greater variability being observed between sessions.

## Materials and Methods

### Subjects

At least 15 volunteers were recruited for each decade of age between 20 and 80 (51 men and 41 women); age ranged from 22 to 77 years. Eighty-three subjects were right-handed and nine left-handed as assessed by self-reporting. None had any history of neurological disorders or diabetes mellitus, and none took any neurotropic medication.

### Ethics Statement

All subjects provided written informed consent. The study was approved by the research ethics committee of Newcastle University’s Medical Faculty (approval number 000023/2008), and conformed to the Declaration of Helsinki.

### Recording

Every effort was made to maintain subjects at a constant level of alertness, and all assessments were carried out on the dominant side. Subjects were seated in a comfortable chair with their arm resting semi-pronated on a cushion. Surface EMG was recorded from first dorsal interosseous (FDI), flexor digitorum superficialis (FDS) and extensor digitorum communis (EDC) in the upper limb, and extensor digitorum brevis (EDB), tibialis anterior (TA) and medial gastrocnemius (MG) in the lower limb. Adhesive electrodes (Bio-Logic M0476; Natus Medical, Mundelein, IL, USA) were placed in a belly-tendon montage over the intrinsic muscles of the hand or foot; for the long muscles of the forearm or calf, the electrodes were placed 4cm apart, one third along the muscle from its proximal origin. Signals were amplified, band-pass filtered (30Hz-2kHz; Digitimer D360, Digitimer, Welwyn Garden City, UK) and digitised at 5kHz (Micro1401, Cambridge Electronic Devices, Cambridge, UK).

### Experiment 1: IMC

In the upper limb, subjects were asked to perform a repetitive precision grip task. A length of compliant plastic tubing (length 19cm, Portex translucent PVC tubing 800/010/455/800; Smith Medical, Ashford, UK) was attached to the index finger and thumb with Micropore tape (3M Health Care, Neuss, Germany), and subjects were asked to oppose both ends of the tubing when prompted by visual and auditory cues. This auxotonic task–so-called because force increases with displacement in a spring-like fashion–required a minimum force of 1N [35] and was similar to a precision grip task used in our previous studies, albeit without measuring digit displacement [36,37]. In the lower limb, subjects were asked to dorsiflex ankle and toes in the air while resting the heel on the ground. Subjects produced 4s of contraction alternating with 2s of relaxation, and at least 100 repetitions. Visual feedback of raw EMG traces was provided to facilitate consistent task performance.

### Experiment 2: IMC Repeated after One Year

After a period of at least one year, eight subjects were asked to repeat the above experiment (six men and two women). Age ranged from 24 to 52 years. All subjects were right-handed.

### Analysis

Analysis was performed in Matlab (Mathworks, Natick, MA, USA) using custom scripts.

Raw data were visually inspected and the first 100 adequately performed trials examined further. Analysis focussed on the early hold phase of the contraction where beta-band oscillations are known to be maximal [6,38]. EMG signals were full-wave rectified. Starting 0.8s after the cue prompting contraction, two contiguous 0.82s-long sections of data from each trial were subjected to a 4096-point fast Fourier transform, giving a frequency resolution of 1.22Hz. Many subjects showed a drop-off in EMG activity so the last 1.56s of the 4s active phase did not enter the analysis. Denoting the Fourier transform of the l^th^ section of the first EMG signal as F_1,l_(λ), the auto-spectrum is given by

$$f_{11}\left(\lambda \right)=\frac{1}{L}\sum_{l=1}^{L}F_{1,l}\left(\lambda \right)\bar{F_{1,l}\left(\lambda \right)}$$

where λ is the frequency (Hz), L is the total number of sections and where the overbar denotes the complex conjugate. For most subjects L was 200; in some individuals fewer than 100 trials remained for analysis after manual review and L was correspondingly lower, with a minimum L of 188 in the upper limb and 174 in the lower limb. The cross-spectrum for two EMG signals with Fourier transforms F_1,l_(λ) and F_2,l_(λ) was calculated as

$$f_{12}\left(\lambda \right)=\frac{1}{L}\sum_{l=1}^{L}F_{1,l}\left(\lambda \right)\bar{F_{2,l}\left(\lambda \right)}$$

Coherence was computed as the cross-spectrum normalised by the auto-spectra

$$C\left(\lambda \right)=\frac{{\left|f_{12}\left(\lambda \right)\right|}^{2}}{f_{11}\left(\lambda \right)f_{22}\left(\lambda \right)}$$

Coherence was calculated for the muscle pairs EDC-FDI, FDS-FDI, MG-EDB and TA-EDB. The wide anatomical spacing between the paired muscles minimised the risk of volume conduction causing inflated coherence values [39].

Under the null hypothesis of linear independence between the signals, a level of significant coherence was determined as [40]

$$Z=1-\alpha^{1/\left(L-1\right)}$$

where the significance level α was set at 0.05.

Analyses of coherence described below were conducted separately for each muscle pair.

To provide a group summary, coherence spectra were averaged across all subjects. The significance level for coherence spectra averaged across the group, or for coherence averaged across the 15-30Hz window in each subject, was determined using the method described by Evans and Baker [41].

In each subject, coherence was averaged across the 15-30Hz window. Log-transformed 15-30Hz coherence was plausibly normally distributed within each decade of age (Shapiro-Wilk test, P≥0.013 for all groups, Bonferroni-corrected significance level α/n = 0.05/(4*6) = 0.0021). However, for the lower limb there was an indication that a normal distribution was not an ideal fit since 8 out of 12 groups had an uncorrected significance level of <0.1, a result which itself has a probability of <0.001 as calculated using the binomial distribution (P = 1-F(7), where F is the cumulative distribution function of B(12,0.1)). In addition, larger samples derived by pooling coherence across all ages (see below) could not be modelled adequately with a normal distribution (Shapiro-Wilk test, P<0.001 in at least one muscle pair for pooled normal data, Bonferroni-corrected significance level α/n = 0.05/4 = 0.0125). Therefore, we chose to model coherence distributions non-parametrically. The variable kernel method adapts the amount of smoothing to the local density of the data [42] and estimates the probability density function (PDF) as

$$\hat{f}\left(x\right)=\frac{1}{N}\sum_{n=1}^{N}\left[\frac{1}{hd_{n,k}}\cdot \phi \left(\frac{x-X_{n}}{hd_{n,k}}\right)\right]$$

where x denotes the log-transformed independent variable, X_n_ the n^th^ log-transformed observation out of a total N observations, d_n,k_ the distance from X_n_ to its k^th^ nearest neighbour, h the global smoothing parameter and ϕ the standard normal PDF. To understand this intuitively: each observation X_n_ was convolved with a Gaussian kernel with a unit area under the curve as specified inside the square brackets, and the sum of these kernels was normalised by N to yield $\hat{f}\left(x\right)$. The window width of the kernel centred on a given observation X_n_ was proportional to d_n,k_ so that broader kernels were associated with observations in regions with sparse data. For any fixed k, the amount of smoothing depended on the global smoothing parameter h; k was set as $\sqrt{n}$ rounded to the nearest integer as suggested by Silverman [42]. Theoretically optimal methods for calculating h have been described but, for our data, resulted in obvious overfitting. Therefore, h was optimised by eye for several datasets; the resulting values of h were empirically fitted with simple algebraic expressions and the approximation $\sqrt[{12}]{n-5}$ was chosen to determine h subsequently. It should be emphasised that this expression has no theoretical value, but merely provided a convenient shorthand way of determining h objectively for each dataset.

Log-transformed coherence has a bounded domain of (–∞,0]. To ensure that the PDF was zero for x>0, $\hat{f}\left(x\right)$ was modified by reflection in the boundary [42]:

$$\hat{g}\left(x\right)=\{\begin{array}{rr} \hat{f}\left(x\right)+\hat{f}\left(-x\right), & x\leq 0\\ 0, & x>0 \end{array}$$

The resulting PDF still integrated to unity and observations near the boundary retained the same magnitude of contribution to the PDF. The estimated cumulative distribution function was calculated as

$$\hat{G}\left(y\right)=\int_{-\infty }^{y}\hat{g}\left(x\right)dx$$

where y is the log-transformed independent variable.

Log-transformed coherence was compared between all decades of age using a Kruskal-Wallis test, and between individual muscle pairs or limbs using a two-tailed paired t-test.

Coherence measures the amplitude of coupling between two signals, and hence was used in the analysis described above (after log-transformation to better represent the wide range of values). However, when it is desired to compare two coherence measurements made on separate occasions, it is possible to generate a Z-score via a hyperbolic arctan transform which has known statistical properties. To measure changes in coherence occurring during experiment 1 (‘intrasession’), the recording session from each subject was split into two consecutive epochs each comprising the same number of trials. Coherence spectra were estimated separately for each epoch, and the significance of changes between both epochs was determined by calculating single-subject Z-scores as

$$Z_{n_{s}}=\sqrt{\frac{L}{N_{\lambda }}}\sum_{\lambda =\lambda_{1}}^{\lambda_{2}}\left(\text{atanh}\left(\sqrt{C_{n_{s}}^{second}\left(\lambda \right)}\right)-\text{atanh}\left(\sqrt{C_{n_{s}}^{first}\left(\lambda \right)}\right)\right)$$

where $C_{n_{s}}^{first}\left(\lambda \right)$ and $C_{n_{s}}^{second}\left(\lambda \right)$ denote the coherence at frequency λ for subject n_s_ during the first and second epoch. $Z_{n_{s}}$ was summed over all N_λ_ frequency bins in the 15-30Hz window (N_λ_ = bin number(λ_2_)–bin number(λ_1_)+1 = 12), and normalised so that it should be normally distributed with zero mean and unit variance under the null hypothesis of no change in coherence between epochs [40,43].

The compound Z-score across all N_s_ subjects was calculated as

$$\hat{Z}=\sqrt{\frac{1}{N_{s}}}\sum_{n_{s}=1}^{N_{s}}Z_{n_{s}}$$

The associated two-tailed probability (P_Z_) was computed with reference to the standard normal distribution, which tested deviations from the null hypothesis that the two coherence measures were drawn from underlying distributions with identical means.

Single-subject Z-scores should be normally distributed with mean zero and variance one, on the null hypothesis that each measure of coherence in that subject was drawn from the same underlying distribution. We tested whether Z-scores were more variable than expected on this assumption, which would imply a biological process with increasing variability from one measurement to the next rather than merely statistical estimation error. The significance of any difference in the variance of single-subject Z-scores from the unit variance expected under the null hypothesis was estimated using Monte-Carlo simulations. Each simulation involved drawing N_s_ random samples from a standard normal distribution and calculating their variance. This procedure was repeated 10^6^ times, allowing the distribution of the variance to be estimated under the null hypothesis that coherence did not change between epochs. The two-tailed probability for the observed variance (P_MC_) was calculated from this estimated null distribution.

For experiment 2, we calculated both intersession and intrasession variances. Coherence spectra were estimated separately for two epochs per session as described above. Intrasession changes were quantified in each subject by calculating one Z-score, comparing both epochs of session 1, and a second Z-score, comparing both epochs of session 2. The intrasession variance was computed as the variance of all resulting Z-scores, i.e. two Z-scores from each subject. Similarly, intersession changes were measured in each subject by calculating one Z-score, comparing the first epochs of both sessions, and a second Z-score, comparing the second epochs; intersession variance was calculated from all resultant Z-scores.

The significance of the difference between intersession and intrasession variance was estimated by means of Monte-Carlo simulations. For each subject, coherence spectra were shuffled across epochs and sessions, and the difference between intersession and intrasession variance was recalculated. This process was repeated 10^6^ times, allowing the null distribution of the difference in variances to be estimated. The two-tailed probability for the observed difference was computed with reference to the estimated null distribution.

### Results

**Fig 1:**
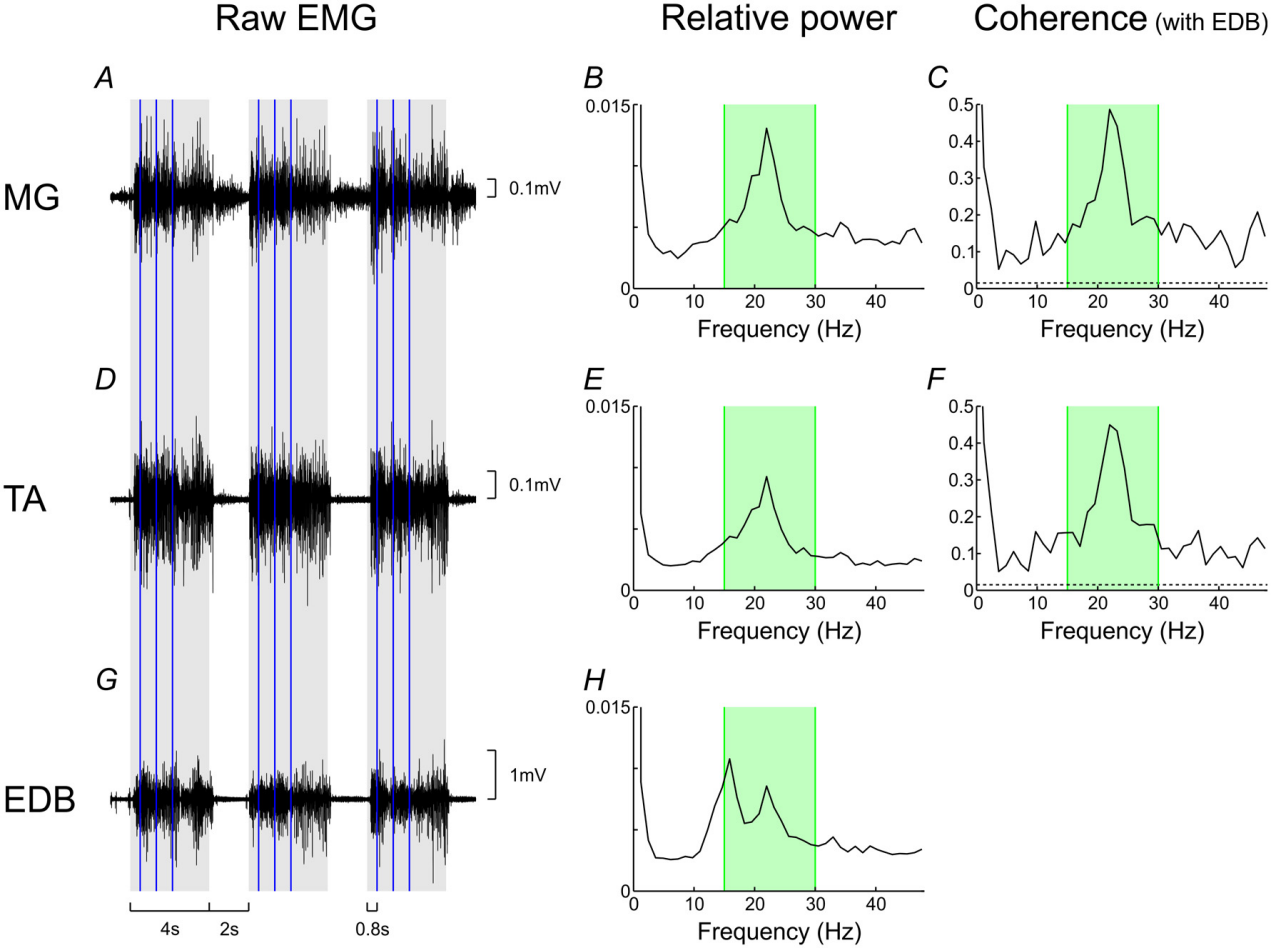
Single-subject power and coherence in the lower limb. Raw EMG is shown for three sample trials (A,D,G). The grey boxes indicate the cued contraction phase of the task, and the vertical blue lines represent the two fast Fourier transform windows during the hold phase. The spectral plots show relative power (B,E,H) and coherence with EDB (C,F). The 15-30Hz beta-band is designated by the green boxes, and the dotted horizontal lines indicate the significance level for coherence. In most subjects, power and coherence spectra peaked in the 15-30Hz band.

**Fig 2:**
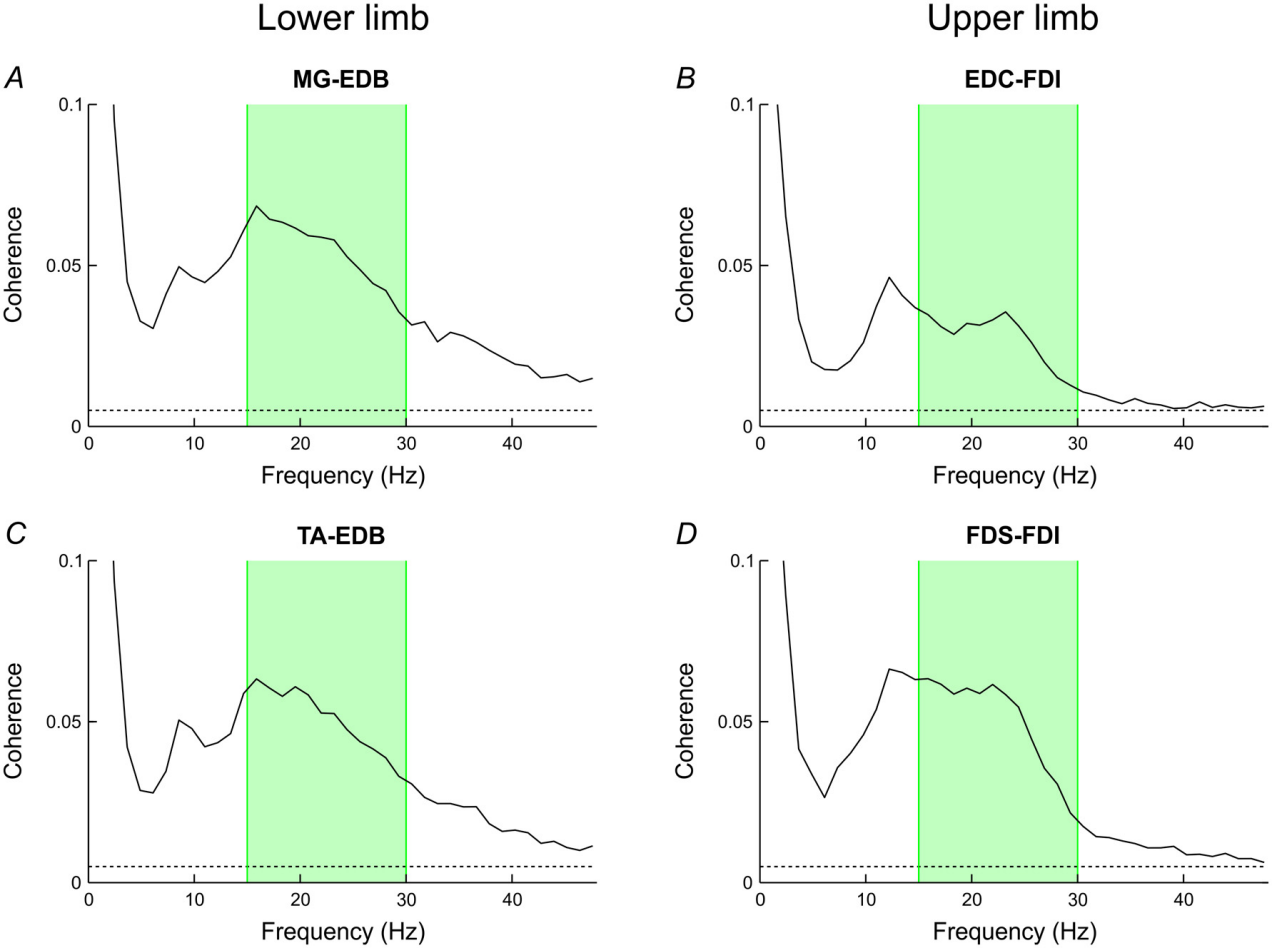
Group data for coherence. Average coherence spectra are shown for MG-EDB and TA-EDB in the lower limb (A,C) and for EDC-FDI and FDS-FDI in the upper limb (B,D). The dotted horizontal lines represent the significance level for average coherence, and the green boxes indicate the 15-30Hz beta-band. Significant average coherence was present in the 15-30Hz band for each muscle pair. Typically, coherence demonstrated a peak or an inflexion within this window and a further one around 9-12Hz whilst dropping off at higher frequencies.

Single-subject data are shown in Fig 1 for the lower limb. In most subjects, power and coherence peaked in the 15-30Hz band. On group averages, coherence in all muscle pairs was significant across the 15-30Hz window and showed either a peak or an inflexion inside this frequency band (Fig 2).

**Fig 3:**
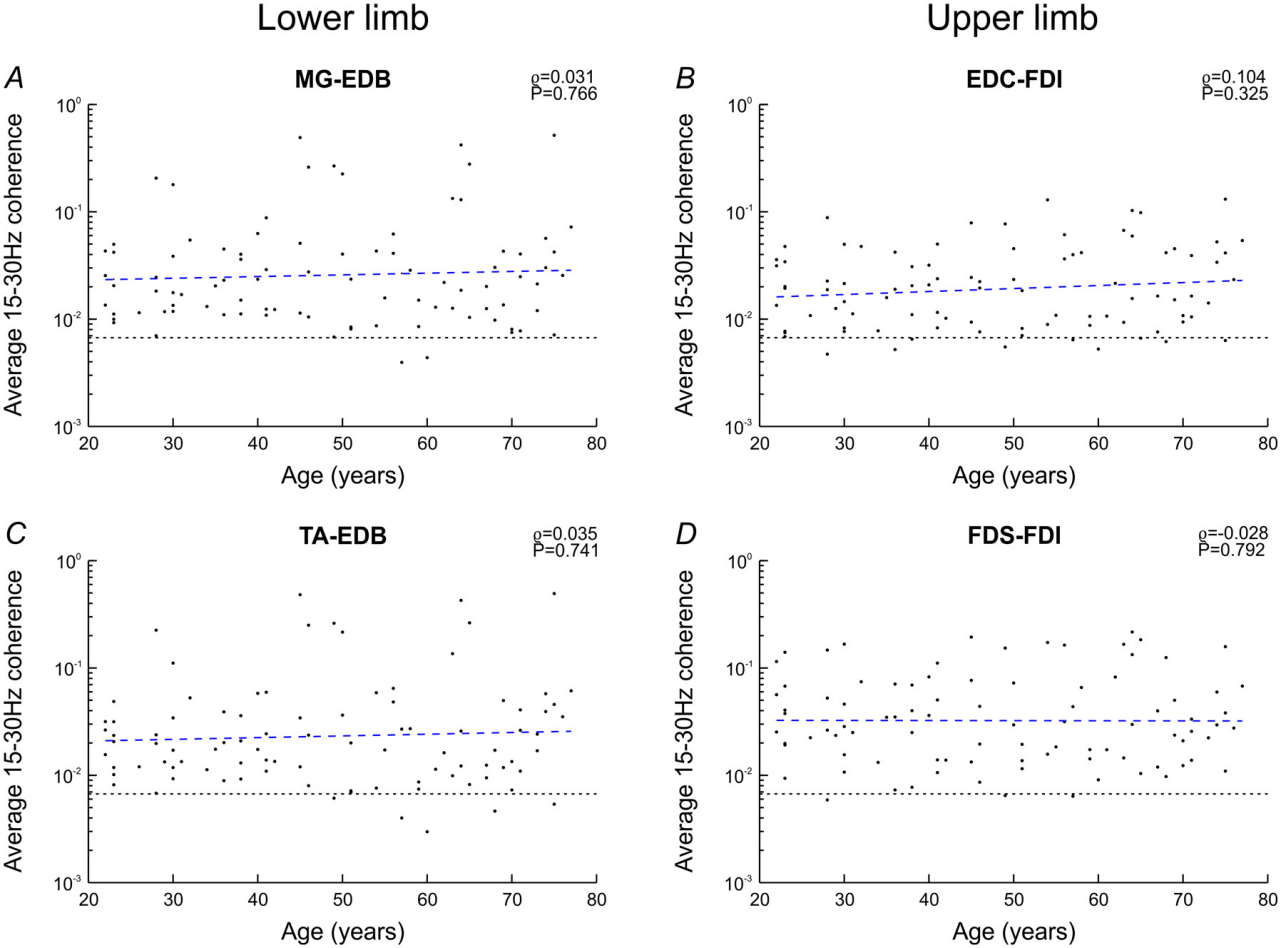
Correlation between coherence and age. For each muscle pair, average 15-30Hz coherence is plotted against age. There was no significant correlation between coherence and age in any muscle pair (Spearman’s ρ). Linear regression models are shown as the blue dashed lines, and significance levels for average 15-30Hz coherence (for L = 200) are represented by the horizontal dotted lines.

Coherence was averaged across the 15-30Hz window in each subject; for brevity average 15-30Hz coherence is referred to as ‘coherence’ from here on. There was no significant correlation between coherence and age in any muscle pair (Spearman’s ρ≤0.104, P≥0.325; Fig 3), with interindividual variability spanning up to two orders of magnitude. Coherence was non-significant in EDC-FDI in 9 subjects, in FDS-FDI in 3 subjects, in MG-EDB in 2 subjects, and in TA-EDB in 5 subjects. The significance level shown in Fig 3 and subsequent Figures is based on L = 200. However, use of individualised significance levels based on each subject’s respective L resulted in the same number of subjects with non-significant coherence in each muscle pair.

**Fig 4:**
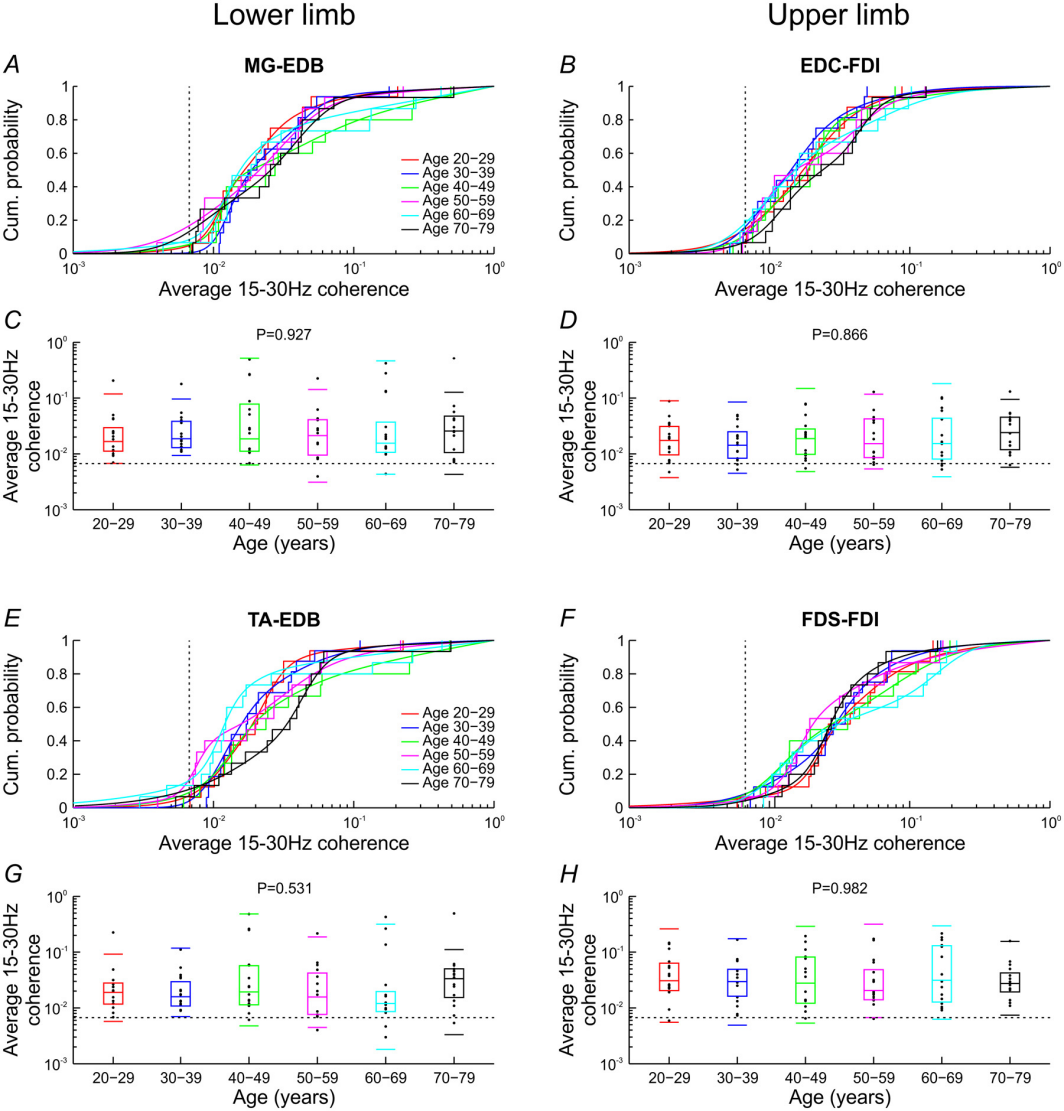
Coherence by decade of age with corresponding variable kernel density estimates and summary statistics. Each decade is illustrated in a different colour. The stairstep curves show the distribution of average 15-30Hz coherence for subjects within a given decade, with the smooth curves showing corresponding density estimates (A,B,E,F). Quartiles derived from density estimates are shown by the box plots with additional horizontal lines indicating 5^th^ and 95^th^ centiles, overlain on a dot plot of individual coherence values (C,D,G,H). Coherence did not vary significantly with age (Kruskal-Wallis test). Significance levels for average 15-30Hz coherence (for L = 200) are shown as the vertical or horizontal dotted lines.

The distribution of coherence was similar for all decades of age as illustrated by the stairstep curves in Fig 4A, 4B, 4E and 4F. The smooth curves show the corresponding variable kernel density models. Summary statistics derived from these models are illustrated by the boxplots in Fig 4C, 4D, 4G and 4H, superimposed on dot plots of individual coherence values. Coherence did not vary significantly between decades (Kruskal-Wallis test, P≥0.531).

**Fig 5:**
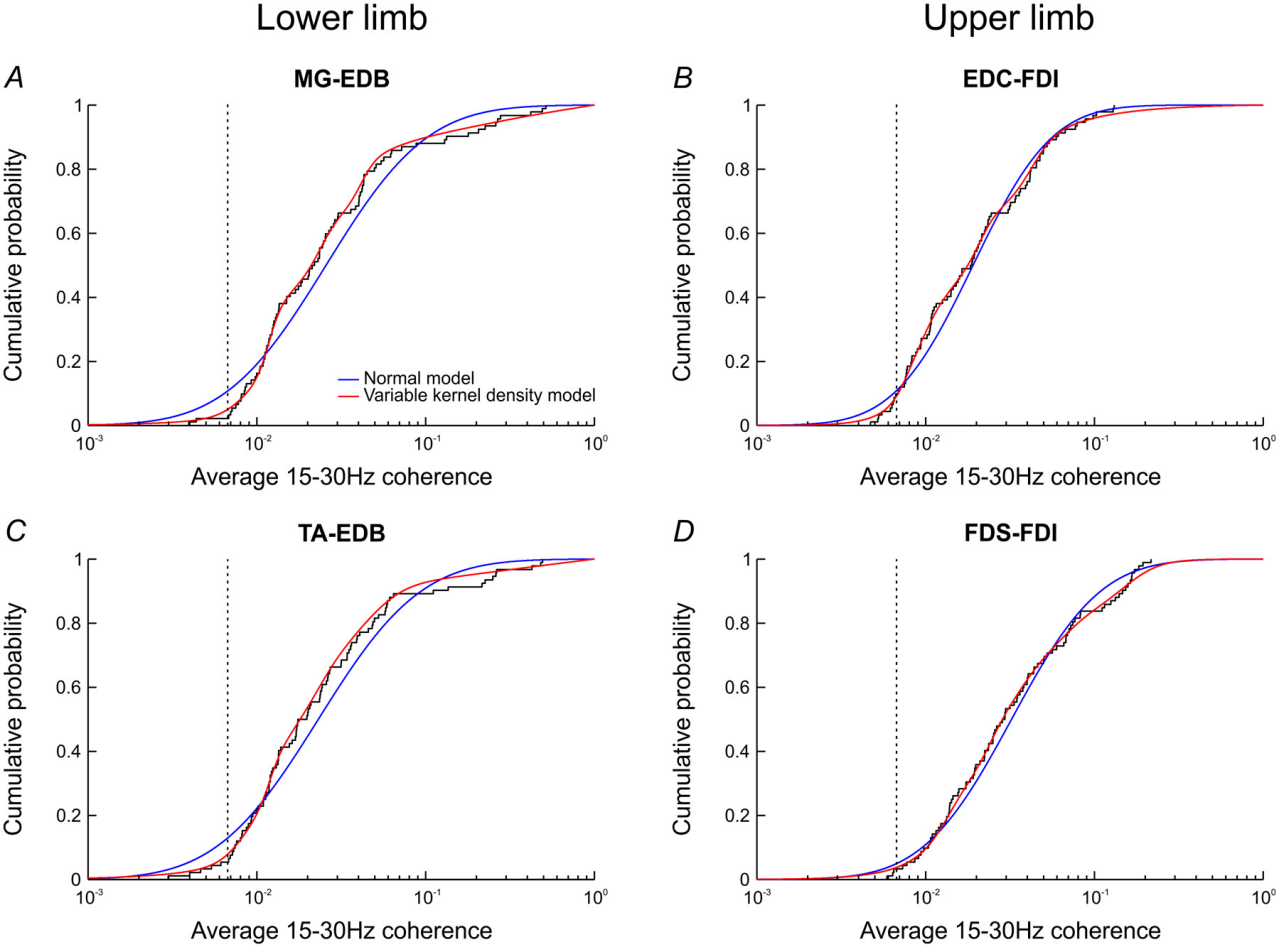
Coherence across all ages with normal and variable kernel density models. Since coherence did not vary with age, we pooled coherence readings across all ages into a single dataset. The stairstep curves illustrate the distribution of average 15-30Hz coherence for subjects of all ages. The data were modelled with a normal distribution (blue) and variable kernel density estimation (red). The density estimation model achieved a closer fit throughout, whilst still smoothing out some of the variability of the data. Significance levels for average 15-30Hz coherence (for L = 200) are shown as the vertical dotted lines.

Because there was no dependence on age, we pooled coherence values across all ages into a single dataset (Fig 5). The combined dataset was modelled empirically with a normal distribution (blue) and a variable kernel density estimate (red). The latter achieved a closer fit throughout whilst still smoothing out much of the small-scale variability of the data. We propose this cumulative distribution as a normative dataset for healthy subjects, against which future experimental findings can be compared. Individual beta-band coherence averages and a table of the cumulative distribution functions resulting from variable kernel density estimation are available from the CARMEN repository.

Log-transformed coherence was significantly greater in FDS-FDI than in EDC-FDI (two-tailed paired t-test, P<0.001) and in MG-EDB than in TA-EDB (P<0.001). By contrast, there was no significant difference in log-transformed coherence between upper and lower limbs (P = 0.596).

**Fig 6:**
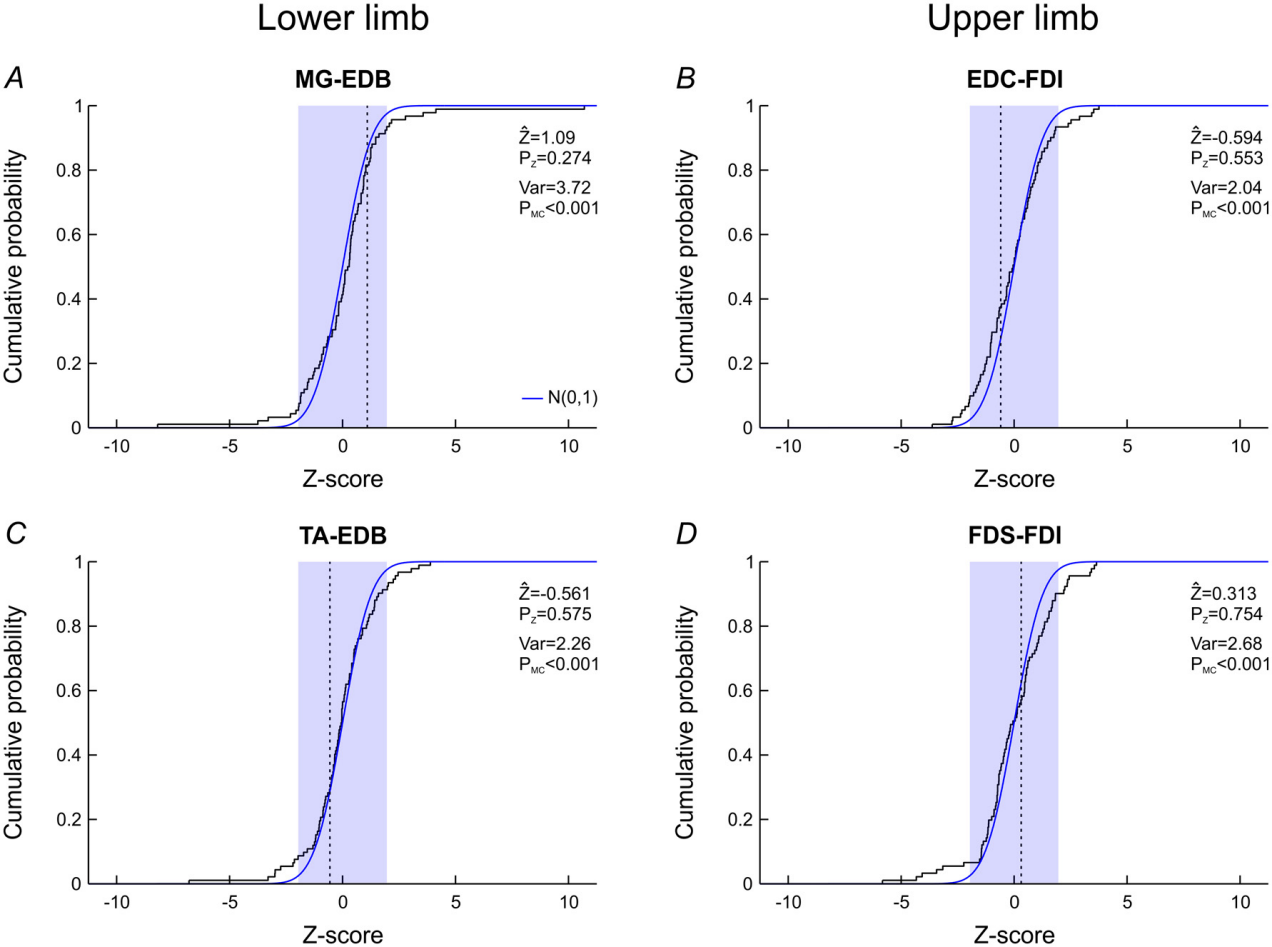
Z-scores for intrasession differences in coherence. In each subject, the recording session was divided into two epochs for which separate 15-30Hz coherence values were calculated. Single-subject Z-scores quantify the difference between both coherence values in each individual and their distribution is shown by the stairstep curves; under the null hypothesis, they should follow a standard normal distribution (blue curve). The mean compound Z-score for all subjects ($\hat{Z}$; vertical dotted lines) was not significant in any muscle pair (P_Z_; blue box showing range of ±1.96). However, in all muscle pairs the variance of individual Z-scores (Var) was significantly greater than unity as estimated by Monte-Carlo simulations (P_MC_).

In order to assess the stability of coherence within a recording session (‘intrasession’), we determined single-subject Z-scores for differences in coherence between two halves of the same session (Fig 6). For all muscle pairs, the mean compound Z-scores were not significantly different from zero (P_Z_≥0.274) but the variances of single-subject Z-scores were significantly greater than unity (P_MC_<0.001). Thus, coherence showed greater variability within a recording session than would be statistically expected.

**Fig 7:**
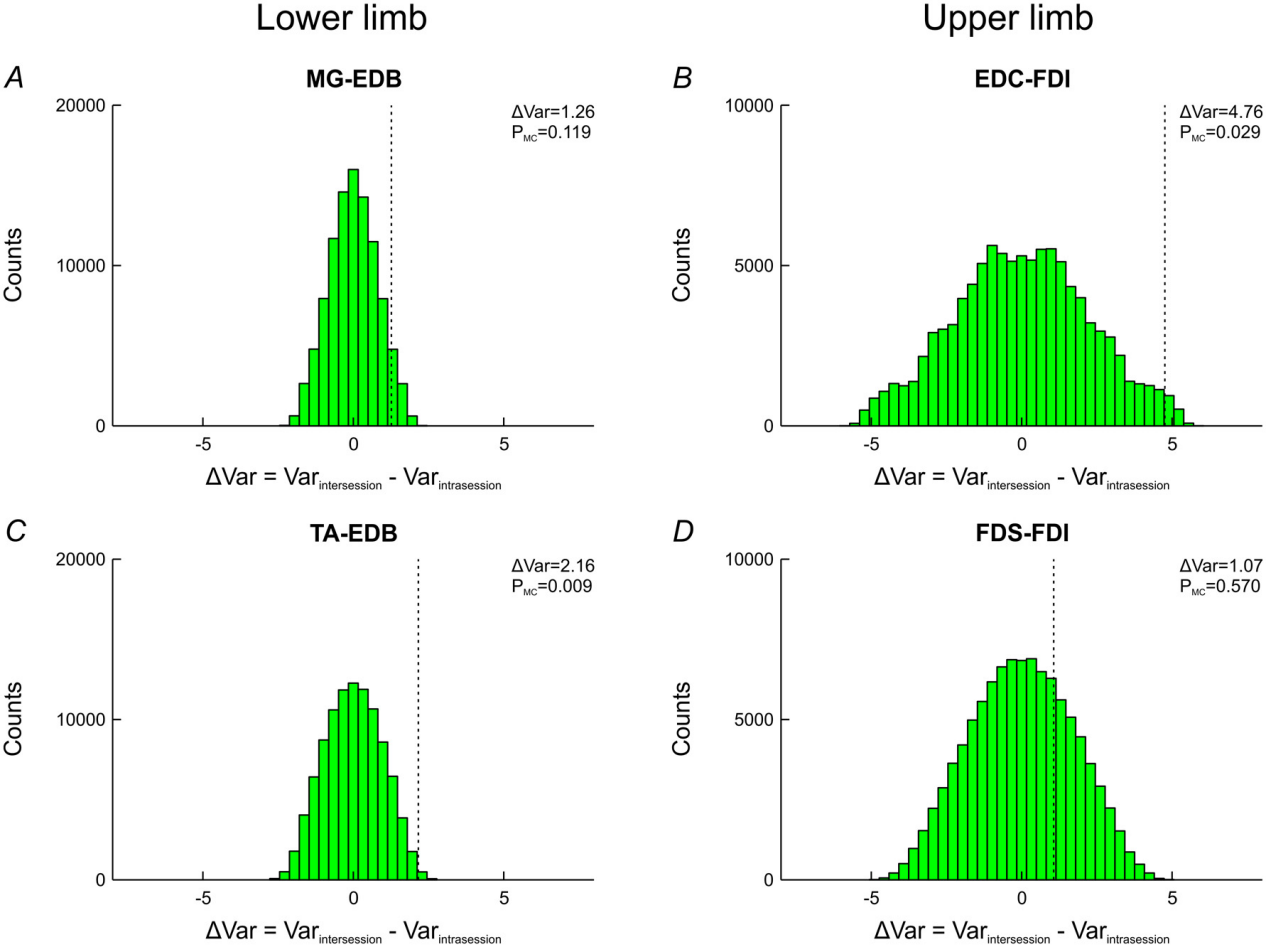
Difference between variances of intersession and intrasession Z-scores. Each recording session from experiment 2 was split into two epochs for which separate 15-30Hz coherence values were computed. Single-subject Z-scores were calculated for differences in coherence between both halves of the same session (‘intrasession’) and for differences in coherence between corresponding halves of both sessions (‘intersession’). The difference of the variances of intersession and intrasession Z-scores was then computed (ΔVar; vertical dotted lines). For each muscle pair, the null distribution was estimated using Monte-Carlo simulations (green histograms). The probability of the observed value occurring under the null hypothesis was calculated with reference to the estimated null distribution (P_MC_). Intersession variance exceeded intrasession variance in all muscle pairs, with the differences reaching significance in TA-EDB and EDC-FDI.

A subset of the cohort returned after one year for a second recording session, allowing us to investigate whether coherence exhibited greater variability between than within sessions. We calculated single-subject Z-scores for differences in coherence between both halves of the same session (‘intrasession’) and for differences in coherence between corresponding halves of both sessions (‘intersession’). In all muscle pairs, the intersession variance of single-subject Z-scores was greater than the intrasession variance (Fig 7), with the differences reaching significance in TA-EDB (P_MC_ = 0.009) and EDC-FDI (P_MC_ = 0.029).

## Discussion

Beta-band IMC did not change significantly across almost six decades of adulthood. There was considerable between-subject variability; within a given subject and session, variability was larger than statistically expected, and between sessions variability was even greater.

IMC requires only a single recording modality and is present more consistently than CMC in healthy subjects [44]. Our task involved minimal instrumentation and weak, phasic contractions, optimising applicability of IMC in patients with neurological deficits.

### IMC in Adulthood

Our finding of a lack of significant change in beta-band IMC amplitude throughout adulthood agrees with two previous studies describing no significant differences in CMC [13] or IntraMC amplitude [21] between young and old adults. Whilst another study reported an increase in CMC with age, significance levels were borderline (P = 0.04) [20]. The weight of the evidence therefore suggests that coherence amplitude remains unchanged throughout adult life.

Senescent alterations in the efferent-afferent feedback loop might be expected to disrupt beta-band coherence. There is strong evidence that peripheral nerve conduction velocities decline with age [31,32]. Whilst central conduction velocities appear to remain constant [45,46], ageing is associated with morphological changes in the central sensorimotor pathways [29,30] and may alter transmission in a manner which is not detectable on motor or sensory evoked potentials. Beta-band coherence is thus surprisingly robust to alterations in conduction pathways, and this is supported by two observations unrelated to old age: IMC reaches adult values in early teenage although peripheral conduction times continue to increase with limb length for several years [14]; and beta-band coherence is present in primates of different sizes despite marked size-related differences in conduction delays [6].

Reduced intracortical inhibition in old age [28] might be expected to lead to diminished coherence, but no such change was observed. It is possible that intracortical inhibition is critical to the initial patterning of the corticospinal drive but has a less prominent role in maintaining oscillatory activity in the efferent-afferent feedback loop. One study boosted intracortical inhibition in adults using diazepam, a GABAergic agent. The power of 15-30Hz oscillations on the electroencephalogram increased yet CMC amplitude decreased [43]. These results highlight that coherence can be dissociated from alterations in cortical oscillatory activity, suggesting that that the relationship between intracortical inhibition and coherence might not be straightforward.

One aim of this study was to gather a normative dataset for potential clinical applications. Since IMC across the 15-30Hz band appears to be related to the integrity of the corticospinal tract, we focussed on average 15-30Hz IMC as our summary measure, thus also ensuring comparability with our previous study [35]. By contrast, most past reports analysed the amplitude and frequency of peak coherence in the 15-30Hz window [12,13,20,34]. Coherence estimates include a noise component and do not always show a clear single peak. Our approach of averaging across N_λ_ = 12 frequency bins circumvented any difficulties in quantifying single or multiple peaks and boosted signal-to-noise ratio by $\sqrt{12}\approx 3.5$. We did not analyse coherence outside the 15-30Hz window as it is less clearly associated with the corticospinal tract. At lower frequencies, potential generators of coherence include the reticular formation, cerebellum and local spinal circuits [47]. At higher frequencies, coherence may involve neural substrates other than the corticospinal tract and only becomes prominent during tasks involving strong [48] or dynamically modulated contractions [49].

The lack of age-related changes allowed us to pool results, thus maximising the effective size of the control group for studies of IMC in neurological conditions. The aggregate dataset could not be fitted adequately by a normal distribution or simple empirical formulae. Whilst variable kernel density estimation provided a good fit, it did not allow the data to be summarised using a small number of numerical parameters.

### Variability of IMC

Interindividual variability of IMC ranged over two orders of magnitude. The degree of variability did not appear to change with age, militating against the possibility that undiagnosed neurological issues in older subjects could have caused a greater spread of coherence readings. Other than genuine differences between subjects, sources of variability within individuals may also have played a role.

Intrasession variance of IMC was significantly greater than predicted by statistical variation alone. Two potential causes are fluctuations in task performance and random moment-to-moment variation of central coupling mechanisms, which might be apparent even after the averaging process inherent in coherence analysis. Previous studies employed tasks which can be readily standardised but require prolonged activity or a high level of dexterity and thus are not suitable for use in patients with neurological deficits. Our task involved weak, phasic contractions and a minimum of instrumentation. Inherent disadvantages were a greater freedom of movement and less precisely defined targets, making it more difficult to ensure consistent performance; further task optimisation might be possible to reduce variability. Random variation of coherence is seen during sustained contractions in normal subjects. Periods of increased coherence are thought to promote sensorimotor recalibration at the expense of increased 15-30Hz tremor and delayed reaction times [50,51]. Such variation contributes to the overall variability of coherence and appears to be neither consciously controlled nor related to specific features of the task.

Intersession variance was greater than intrasession variance, reaching significance in two muscle pairs. Two aspects may have contributed in addition to the above factors. Firstly, it is not possible to achieve an identical electrode montage after one year. In a previous study, electrode position significantly influenced coherence between two intrinsic hand muscles [52], but the proximity of the two muscles means that electrical cross-talk may have confounded the results. It would be useful to re-address this issue in a muscle pair with greater separation. Secondly, coherence amplitude and directionality can change substantially within an individual over a timeframe of one or two years, even with a highly instrumented task [11]. The timeframe of these changes should be clarified by obtaining multiple coherence estimates at shorter intervals.

## Conclusion

This is one of the largest studies of beta-band coherence in healthy adults to date. IMC showed no significant age-related changes across almost six decades of age; therefore results were pooled across all ages into a combined normative dataset.

Interindividual variability of IMC spanned two orders of magnitude. Intrasession variance was significantly greater than statistically expected; potential reasons include moment-to-moment variability of central coupling and changes in task performance. In a smaller cohort, we examined intersession variance for two sessions separated by one year and found even greater variability. Additional causes include longer-term changes in coherence within individuals and differences in electrode montage. Limiting the variability of IMC measurements in normal subjects is critical to future clinical applications. Significant reductions should be achieved by experiments aimed at task optimisation.
